# Deep inspiration breath-hold reduces esophageal dose in spinal stereotactic body radiotherapy: A feasibility study

**DOI:** 10.1016/j.tipsro.2025.100358

**Published:** 2025-11-29

**Authors:** Takahiro Aoyama, Yutaro Koide, Tomoki Kitagawa, Ryoma Tomoda, Shingo Hashimoto, Hiroyuki Tachibana, Takeshi Kodaira

**Affiliations:** Department of Radiation Oncology, Aichi Cancer Center, 1-1 Kanokoden, Chikusa-Ku, Nagoya, Aichi 464-8681, Japan

**Keywords:** Spinal stereotactic body radiotherapy (SBRT), Deep inspiration breath-hold (DIBH), Esophageal dose reduction, Vertebral–esophageal distance, Re-irradiation

## Abstract

We aimed to assess whether deep inspiration breath-hold (DIBH) could reduce esophageal dose by spinal stereotactic body radiotherapy (SBRT). In 25 patients with thoracic DIBH CT, the vertebral–esophageal distance significantly increased at T2–T9, greatest at Th7 (median 2.1 mm). Simulated SBRT plans showed a strong correlation between distance and esophageal dose, each 1 mm increase reducing 150 cGy. DIBH reduced the esophageal D_0.03 cc_ dose by a median of 411 cGy while maintaining positional reproducibility within 2 mm. These findings suggest that DIBH may reduce esophageal dose and warrants further study in patients with prior high-dose esophageal exposure.

## Highlights

• Spine SBRT with DIBH significantly increased vertebral–esophageal distance at thoracic levels.
• The largest displacement was observed at Th7, with a median increase of 2.1 mm.
• Esophageal dose strongly correlated with vertebral–esophageal distance in SBRT plans.
• DIBH reduced esophageal D_0.03cc_ dose by 411 cGy without compromising target coverage.
• Findings suggest DIBH may allow safer re-irradiation for patients with prior high-dose exposure.

## Introduction

Spinal stereotactic body radiotherapy (SBRT) is a treatment technique that enables the delivery of high radiation doses to spinal tumors while reducing radiation exposure to the spinal cord. In a phase III trial comparing SBRT with conventional radiotherapy for pain palliation, SBRT demonstrated superiority in the rate of complete pain relief at 3 months [1]. Furthermore, patients treated with SBRT showed a significantly lower local recurrence rate at 1 year and a reduced need for re-irradiation, suggesting that SBRT may offer advantages in long-term tumor and pain control [2]. Building on these favorable outcomes, SBRT has also been investigated as a promising option for re-irradiation in previously treated patients [3].

On the other hand, concerns have been raised regarding the increased risk of adverse events following SBRT, including pain flare and vertebral compression fractures [[4], [5], [6]]. In particular, severe complications, even fatal events, have been reported in patients with a history of high-dose irradiation to the esophagus, highlighting the necessity of strict adherence to dose constraints. The ESO-SPARE trial reported that esophageal-related adverse events occurred in over half of the patients receiving palliative spine RT (64 % in the standard arm) and found a correlation between high esophageal dose and the risk of “severe-or-worse” dysphagia [7]. Furthermore, Koide et al. reported a treatment-related death (Grade 5) due to esophageal perforation in a re-irradiation SBRT patient who had received high-dose prior conventional RT [8]. Conversely, there is solid evidence that SBRT is safe when established esophageal dose constraints are satisfied, and strict adherence to these constraints is crucial. However, because the vertebrae and esophagus are often anatomically adjacent, achieving sufficient target coverage while limiting esophageal dose remains a clinical trade-off. In current clinical practice with intensity-modulated radiation therapy, this often necessitates compromising the coverage of the anterior vertebral body to meet the esophageal constraint, which is a major concern when the gross tumor volume is in the anterior vertebral body or if there is soft tissue tumor extension anteriorly from the spine. Therefore, new technical strategies are required to reduce esophageal dose without compromising treatment intensity or efficacy.

Deep inspiration breath-hold (DIBH) has been widely implemented as a method to maintain treatment efficacy while reducing radiation dose to surrounding normal tissues. By holding the breath at deep inspiration, the heart can be displaced away from the irradiation field, and its clinical benefits have been reported in numerous studies [9]. Recently, DIBH has also been applied in SBRT for the lung and liver, allowing for reduced dose to normal tissues while preserving target coverage [10]. In particular, the previous report by Giraud et al. [11] showed that the dosimetric advantages obtained with DIBH correlated with a significant reduction in late esophageal toxicities.

Based on this background, we hypothesized that in spinal SBRT, DIBH could achieve esophageal dose reduction by increasing the distance between the vertebral body and the esophagus as a result of thoracic volumetric expansion during deep inspiration. The aim of the study is to investigate if using DIBH can increase the vertebral-esophagus distance and reduce dose to the esophagus in spine SBRT.

## Materials and methods

### Study design and data sources

This retrospective study was approved by the Institutional Review Board (approval number: 2024–0-519) and conducted in accordance with relevant guidelines and regulations. Twenty-five female patients who had undergone DIBH procedures for adjuvant breast radiotherapy at our institution between 2018 and 2024 were randomly selected, and simulated SBRT plans were generated for the spine. The CT acquisition protocol for DIBH followed previously published methods [12], and all patients successfully completed the DIBH procedure according to the institutional protocol. For each patient, three series of DIBH CT images and one series of FB images were obtained, resulting in a total of four CT datasets per patient. Patients were positioned supine with arms raised above the head on an upper-limb support device. Neither thermoplastic masks nor vacuum cushions were used during imaging.

### Endpoints

The primary endpoint was to evaluate the extent to which DIBH could reduce the esophageal dose. Specifically, vertebral–esophageal distances were compared between DIBH and FB, and the vertebral levels showing the greatest benefit from DIBH were identified. In addition, from the simulated SBRT plans, the relationship between vertebral–esophageal distance and esophageal dose was analyzed to quantify the dose-sparing effect of DIBH. Secondary endpoints included the reproducibility of vertebral position across multiple DIBH acquisitions and the beam-on time during DIBH.

### Evaluation of vertebral–esophageal distance

In all CT datasets (three DIBH and one FB), the thoracic vertebrae (ThX) and esophagus were contoured. Contouring of the thoracic vertebrae and esophagus was performed manually by a single radiation oncologist. The final contours were verified through peer review by two other certificated radiation oncologists. The shortest three-dimensional distance between each vertebra and the esophagus was automatically calculated using a Python script within the treatment planning system and defined as the vertebral–esophageal distance. For DIBH, the minimum value among the three acquisitions was used for analysis. Statistical comparison of distances between DIBH and FB was performed using the Wilcoxon signed-rank test. The vertebral level with a significant difference and the greatest median increase in distance was defined as the most effective level for DIBH.

### Treatment planning

At the vertebral level showing the greatest increase in vertebral–esophageal distance with DIBH, simulated SBRT treatment plans were generated. The clinical target volume (CTV) was defined as the vertebral body, and a 2-mm isotropic margin was added to create the planning target volume (PTV). An evaluation structure (PTV_evaluate) was defined as the PTV minus the thecal sac. For DIBH, contours were delineated on all three DIBH datasets, and the composite structures (e.g., internal CTV) were used for dose evaluation. A prescription dose of 24 Gy in 2 fractions was applied, and dose constraints for targets and organs at risk followed established protocols [3]. Specifically, the esophageal near-maximum dose (D_0.03 cc_) was constrained to less than 20 Gy. Plans were generated using a TrueBeam linear accelerator with a single full arc. Treatment planning was performed using RayStation version 10.0 (RaySearch Laboratories, Stockholm, Sweden) with the collapsed cone convolution algorithm for dose calculation. Optimization calculations were performed by a single medical physicist with experience in over 400 SBRT cases annually, and all treatment contour and plans were approved by two certificated radiation oncologists. The relationship between vertebral–esophageal distance and maximum esophageal dose was analyzed using Spearman’s correlation coefficient. Esophageal maximum doses between DIBH and FB were compared using the Wilcoxon signed-rank test. All statistical analyses were performed using R software, version 3.6.1 (The R Foundation for Statistical Computing, Vienna, Austria). In addition, the estimated median beam-on time (BOT) for DIBH was obtained from the treatment planning system.

### Reproducibility of vertebral position with DIBH

Reproducibility of vertebral position during DIBH was assessed by calculating the inter-series variation (Σ) and intra-patient variation (σ) from the three DIBH image sets. Reproducibility was defined using the following formula [13]:

Reproducibility = 2.5Σ × 0.7σ (mm).

## Results

### Patient characteristics

The baseline characteristics of the 25 patients are summarized as follows: median age was 51 years (range, 27–67). All patients were female with an Eastern Cooperative Oncology Group performance status of 0. All patients had undergone DIBH as part of adjuvant breast radiotherapy, and CT acquisitions were performed according to the institutional protocol.

### Vertebral–esophageal distance

**Fig. 1:**
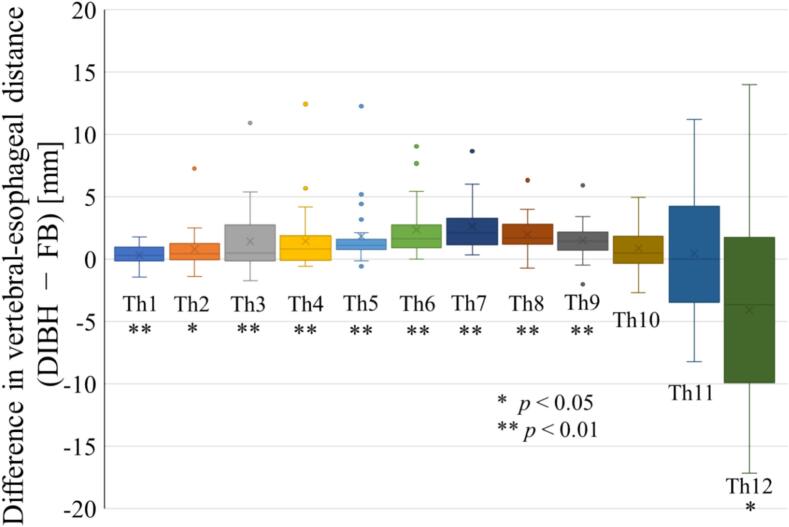
Comparison of vertebral–esophageal distance between deep inspiration breath-hold (DIBH) and free breathing (FB) at each thoracic level.

The vertebral–esophageal distance was compared between DIBH and FB across all thoracic vertebral levels (Fig. 1). DIBH increased the distance at all levels except Th11 and Th12 in the median values. Across Th1–Th10, the change ranged from − 2.0 to + 12.4 mm. In particular, DIBH increased the vertebral–esophageal distance in all patients at Th6 and Th7. Among these, Th7 showed the largest difference, with a median increase of 2.1 mm (range, 0.3–8.6 mm). Based on these findings, Th7 was identified as the vertebral level where DIBH was most effective.

### Esophageal dose reduction

**Fig. 2:**
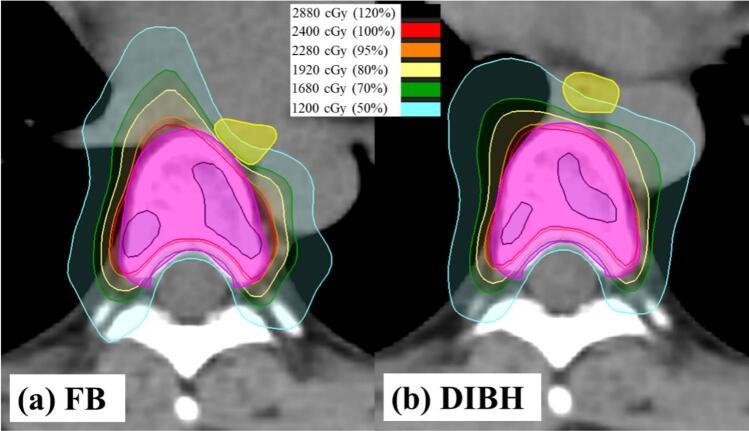
Axial dose distributions of spinal stereotactic body radiotherapy at the seventh thoracic vertebra. (a) Free breathing (FB) plan and (b) deep inspiration breath-hold (DIBH) plan. The pink contour represents the planning target volume, and the yellow contour delineates the esophagus. Compared with FB, DIBH increased the vertebral–esophageal distance, resulting in reduced esophageal dose. (For interpretation of the references to colour in this figure legend, the reader is referred to the web version of this article.)

**Fig. 3:**
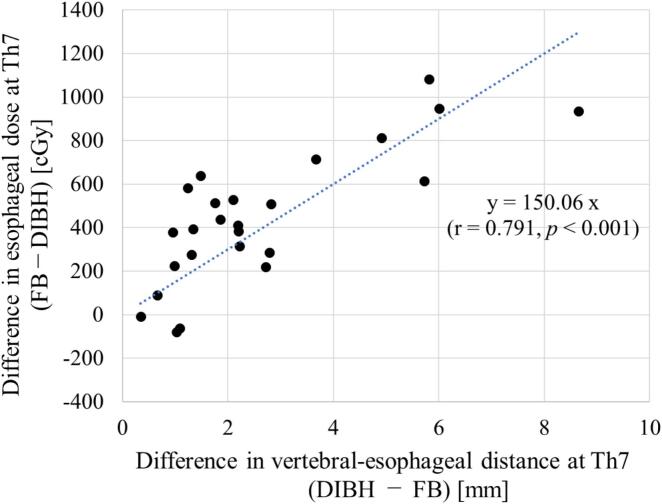
Correlation between vertebral–esophageal distance and maximum esophageal dose in spinal stereotactic body radiotherapy (SBRT) plans at the seventh thoracic vertebra (Th7). FB, free breathing; DIBH, deep inspiration breath-hold.

**Fig. 4:**
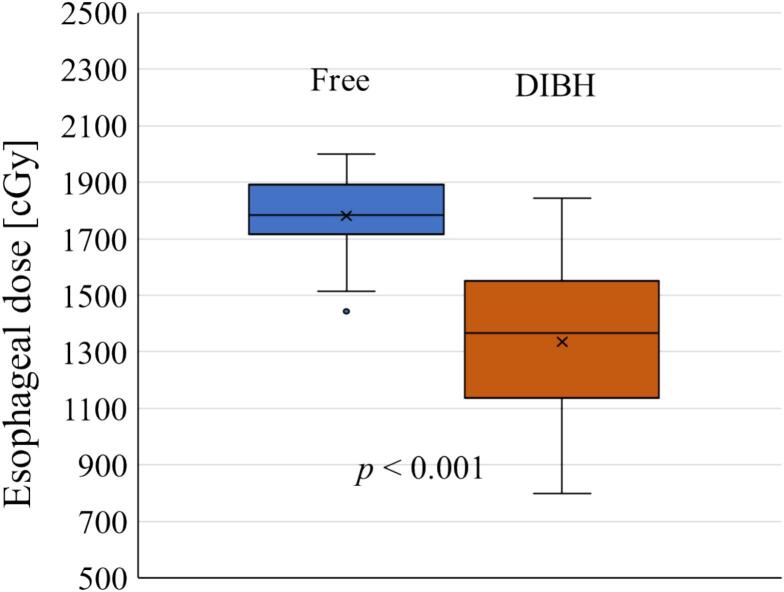
Comparison of maximum esophageal dose between deep inspiration breath-hold (DIBH) and free breathing (FB) at the seventh thoracic vertebra (Th7).

At Th7, simulated SBRT plans were created to evaluate the impact of vertebral–esophageal distance on esophageal dose. Representative axial dose distributions for FB and DIBH are shown in Fig. 2. A strong correlation was observed between the distance and maximum esophageal dose (Spearman r = 0.791, *p* < 0.001), indicating that each 1-mm increase in distance corresponded to an approximate 150 cGy reduction in esophageal dose (Fig. 3). Compared with FB, DIBH significantly reduced the maximum esophageal dose (*p* < 0.001), with a mean reduction of 446 cGy and a median reduction of 411 cGy (range, 80–1081 cGy) (Fig. 4).

### Reproducibility and BOT

From the three DIBH image sets, the reproducibility of Th7 position was calculated as 1.8 mm, which was within the 2-mm setup margin commonly applied in spinal SBRT. However, as intra-fractional variation during breath-hold was not evaluated, this result should be interpreted with caution. The median BOT for DIBH was 99 s (range, 88–110 s).

## Discussion

To our knowledge, this is the first study to demonstrate the potential utility of deep inspiration breath-hold (DIBH) in spinal SBRT. In particular, we found that DIBH increased the vertebral–esophageal distance at Th7 by a median of 2.1 mm and reduced the maximum esophageal dose by approximately 400 cGy on average. Moreover, a strong correlation was observed between vertebral–esophageal distance and esophageal dose, indicating that each 1-mm increase in distance could yield an approximate reduction of 150 cGy in maximum esophageal dose. While the correlation between anatomical distance and dose is a known physical principle, our study is the first to quantitatively demonstrate that DIBH provides a clinically meaningful dosimetric benefit in spine SBRT, quantifying the reduction of approximately 400 cGy (median 411 cGy) by a 2.1 mm increase in distance at Th7.

Esophageal squamous cell carcinoma frequently arises in the mid-thoracic region [14], and initial definitive treatment often involves high radiation doses of approximately 50 Gy [15]. In a previous report for re-irradiation SBRT [8], ESCC constituted 28.6 % of cases with prior high-dose exposure and included a report of fatal esophageal perforation. Previous randomized trials have demonstrated that esophageal dose is correlated with severe dysphagia [6], and our findings suggest that DIBH may further reduce esophageal dose in this region, thereby providing a safer re-irradiation strategy for patients with a history of high-dose exposure. In addition, challenges in meeting esophageal dose constraints while maintaining target coverage at cervical levels persist, suggesting the applicability of DIBH in the lower C-spine may be an important topic for future study.

The reproducibility of vertebral position during DIBH was 1.8 mm, which was within the 2-mm PTV margin commonly applied in spinal SBRT [1,3]. However, it should be noted that intra-fractional motion due to DIBH is not accounted for. Therefore, an additional planning risk volume (PRV) margin for the spinal cord that considers vertebral motion during DIBH irradiation may be required. On the other hand, increasing the PRV margin could compromise dose coverage of the epidural space, a region prone to recurrence [16]. Therefore, DIBH should not be recommended for patients with extensive epidural involvement, such as those with Bilsky grade ≥ 1b.

Another limitation of DIBH is treatment efficiency. The median beam-on time was 99 s. Assuming a treatment process of approximately 20 s of breath-hold followed by a 30-second rest, patients would require 4–5 breath-hold cycles per fraction. This extension of the total treatment time by 120–150 s should be carefully considered, as it may increase the burden on patients. Therefore, for patients with severe pain from spinal metastases and poor performance status (e.g., performance status ≥ 3) or a high pain score (e.g., Numerical Rating Scale (NRS) ≥ 3), DIBH may not be appropriate due to difficulties in maintaining stable breath-holds.

Based on these considerations, DIBH may be most beneficial for selected patients: those with prior high-dose irradiation to the esophagus (≥50 Gy), without epidural extension of disease (e.g., Bilsky grade ≤ 1a), and with sufficient tolerance for breath-holding (e.g., NRS ≤ 2). This benefit is maximized for lesions at the mid-thoracic level (Th6-Th7), where the greatest displacement was observed. However, careful patient selection and clinical judgment by radiation oncologists remain essential when considering the use of DIBH for spinal SBRT.

Several limitations of this study should be acknowledged. First, the patient cohort consisted entirely of women with breast cancer who underwent DIBH as part of adjuvant radiotherapy, and all had favorable performance status. This introduces a selection bias and may limit generalizability. The retrospective nature of the study precluded a detailed analysis of how BMI, osteoporosis, or other thoracic abnormalities might specifically impact DIBH efficacy. Second, not all patients demonstrated meaningful increases in vertebral–esophageal distance with DIBH, and currently there is no reliable method to predict the degree of dose reduction before planning CT acquisition. This may necessitate additional imaging, raising concerns about increased radiation exposure. Finally, inadequate breath-hold performance during treatment could negatively impact therapeutic outcomes. The derived 1.8 mm reproducibility value was based on three DIBH acquisitions within a single simulation session. This does not account for intra-fractional variations, or changes in patient compliance due to anxiety, pain, or general deterioration on the treatment day. For hypofractionated SBRT with its steep dose gradients, a failure to reproduce the breath-hold position compared to the planning CT can lead to a misalignment and potential severe toxicity, including excessive dose to the spinal cord. Therefore, careful patient training and monitoring are essential for safe clinical implementation.

Overall, the results of this study suggest that DIBH may offer potential benefit in selected patients undergoing linac-based spinal SBRT. However, given the small number of patients analyzed and the inclusion of only one treatment technique, further prospective studies are needed to confirm these findings and establish the clinical utility of DIBH in this setting. A future prospective study should particularly target high-risk patients, such as those with prior high-dose exposure to the mid-thoracic region, and be designed to evaluate clinically relevant endpoints, including treatment-related toxicity, inter- and intra-fractional reproducibility of the breath-hold position, and cumulative esophageal dose safety.

## Conclusion

This study demonstrated that the use of DIBH in spinal SBRT increased the vertebral–esophageal distance at Th7 by approximately 2.1 mm (*p* < 0.0001), resulting in a median reduction of 411 cGy (range, 80–1081 cGy) in maximum esophageal dose. However, the need for multiple breath-hold cycles during treatment introduces disadvantages, including additional treatment time and the potential requirement for increased margins to account for positional reproducibility.

Taken together, our findings suggest that DIBH may be a feasible option for carefully selected patients undergoing spinal SBRT—specifically those with a history of high-dose irradiation to the esophagus (≥50 Gy), without epidural extension (Bilsky grade ≤ 1a), and with sufficient ability to maintain stable breath-hold (NRS ≤ 2). While patient selection and clinical judgment remain crucial, this study provides evidence that DIBH has the potential to improve the safety of spinal SBRT in patients with prior high-dose esophageal irradiation by mitigating the risk of severe toxicity and avoiding the compromise of target coverage.

## Funding statement

No funding was received for this study.

## Declaration of competing interest

The authors declare that they have no known competing financial interests or personal relationships that could have appeared to influence the work reported in this paper.

## References

[1] Sahgal A., Myrehaug S.D., Siva S., Masucci G.L., Maralani P.J., Brundage M. (2021). Stereotactic body radiotherapy versus conventional external beam radiotherapy in patients with painful spinal metastases: an open-label, multicentre, randomised, controlled, phase 2/3 trial. Lancet Oncol..

[2] Zeng K.L., Myrehaug S., Soliman H., Husain Z.A., Tseng C.L., Detsky J. (2022). Mature local control and reirradiation rates comparing spine stereotactic body radiation therapy with conventional palliative external beam radiation therapy. Int J Radiat Oncol Biol Phys..

[3] Kita R, Ito K, Machida R, Sekino Y, Nakamura N, Nakajima Y, et al. Randomized phase III study comparing re-irradiation stereotactic body radiotherapy and conventional radiotherapy for painful spinal metastases: Japan Clinical Oncology Group study JCOG2211 (RESCORE study). Jpn J Clin Oncol. 2024. doi:10.1093/jjco/hyae145. Epub ahead of print. PMID: 39431960. 10.1093/jjco/hyae145 39431960

[4] Pan H.Y., Allen P.K., Wang X.S., Chang E.L., Rhines L.D., Tatsui C.E. (2014). Incidence and predictive factors of pain flare after spine stereotactic body radiation therapy: secondary analysis of phase 1/2 trials. Int J Radiat Oncol Biol Phys..

[5] Faruqi S., Tseng C.L., Whyne C., Alghamdi M., Wilson J., Myrehaug S. (2018). Vertebral compression fracture after spine stereotactic body radiation therapy: a review of the pathophysiology and risk factors. Neurosurgery..

[6] Cox B.W., Jackson A., Hunt M., Bilsky M., Yamada Y. (2012). Esophageal toxicity from high-dose, single-fraction paraspinal stereotactic radiosurgery. Int J Radiat Oncol Biol Phys..

[7] Nielsen A.M., Laursen M.R.T., Rechner L.A., Krog S.M., Storm K.S., Ottosson W. (2025). Esophagus-sparing radiotherapy for complicated spinal metastases (ESO-SPARE): a randomized phase III clinical trial. Radiother Oncol..

[8] Koide Y., Haimoto S., Shimizu H., Aoyama T., Kitagawa T., Shindo Y. (2024). Re-irradiation spine stereotactic body radiotherapy following high-dose conventional radiotherapy for metastatic epidural spinal cord compression: a retrospective study. Jpn J Radiol..

[9] Lu H.M., Cash E., Chen M.H. (2000). Reduction of cardiac volume in left-breast treatment fields by respiratory maneuvers: a CT study. Int J Radiat Oncol Biol Phys..

[10] Boda-Heggemann J., Knopf A.C., Simeonova-Chergou A., Wertz H., Stieler F., Jahnke A. (2016). Deep inspiration breath hold-based radiation therapy: a clinical review. Int J Radiat Oncol Biol Phys..

[11] Giraud P., Morvan E., Claude L., Mornex F., Le Pechoux C., Bachaud J.M. (2011). Respiratory gating techniques for optimization of lung cancer radiotherapy. J Thorac Oncol..

[12] Bartlett F.R., Colgan R.M., Carr K., Donovan E.M., McNair H.A., Locke I. (2013). The UK HeartSpare Study: randomised evaluation of voluntary deep-inspiratory breath-hold in women undergoing breast radiotherapy. Radiother Oncol..

[13] van Herk M. (2000). The probability of correct target dosage: dose-population histograms for deriving treatment margins in radiotherapy. Int J Radiat Oncol Biol Phys..

[14] Then E.O., Lopez M., Saleem S., Gayam V., Sunkara T., Culliford A. (2020). Esophageal cancer: an updated Surveillance Epidemiology and End Results database analysis. World J Oncol..

[15] Hulshof M.C.C.M., Geijsen E.D., Rozema T., Oppedijk V., Buijsen J., Neelis K.J. (2021). Randomized study on dose escalation in definitive chemoradiation for patients with locally advanced esophageal cancer (ARTDECO study). J Clin Oncol..

[16] Thibault I., Campbell M., Tseng C.L., Atenafu E.G., Letourneau D., Yu E. (2015). Salvage stereotactic body radiotherapy following in-field failure of initial SBRT for spinal metastases. Int J Radiat Oncol Biol Phys..

